# Harvest Stage Recognition and Potential Fruit Damage Indicator for Berries Based on Hidden Markov Models and the Viterbi Algorithm

**DOI:** 10.3390/s19204421

**Published:** 2019-10-12

**Authors:** Marcos Orchard, Carlos Muñoz-Poblete, Juan Ignacio Huircan, Patricio Galeas, Heraldo Rozas

**Affiliations:** 1 Department of Electrical Engineering, Universidad de La Frontera, Temuco 4811230, Chile; morchard@ing.uchile.cl (M.O.); juan.huircan@ufrontera.cl (J.I.H.); 2 Department of Electrical Engineering, Faculty of Physical and Mathematical Sciences, Universidad de Chile, Santiago 8370451, Chile; hrozas@ing.uchile.cl; 3 Department of Computer Science, Universidad de La Frontera, Temuco 4811230, Chile; patricio.galeas@ufrontera.cl

**Keywords:** berry harvesting stages, Markov chains, Viterbi algorithm, monitoring, fruit damage indicator

## Abstract

This article proposes a monitoring system that allows to track transitions between different stages in the berry harvesting process (berry picking, waiting for transport, transport and arrival at the packing site) solely using information from temperature and vibration sensors located in the basket. The monitoring system assumes a characterization of the process based on hidden Markov models and uses the Viterbi algorithm to perform inferences and estimate the most likely state trajectory. The obtained state trajectory estimate is then used to compute a potential damage indicator in real time. The proposed methodology does not require information about the weight of the basket to identify each of the different stages, which makes it effective and more efficient than other alternatives available in the industry.

## 1. Introduction

Chile is the main exporter of fresh fruit in the Southern Hemisphere (ODEPA), generating 59.3% of the total production [1]. Worldwide, Chile exports more than 75 different species to more than 100 countries around the world, being a leader in the export of table grapes, plums, apples, blueberries and peaches. In this regard, any improvement in productive processes of fresh fruit harvesting for exportation has a significant impact on the national economy. Those improvements should help to efficiently manage the whole chain of the productive process: Crop, harvest, packing and transport to the destination market.

The fruit produced in Chile is mainly harvested manually (hand picking), but this process requires numerous personnel. While the personnel working in harvesting processes are continuously trained, the vast majority of these workers are employed solely during the productive season. The coordination of this activity requires personnel highly trained in the processes of manual harvesting, since the fruit can suffer damage, mainly mechanical. Indeed, authors such as Li et al. [2] state that fresh fruit is susceptible to mechanical damage during the whole process, from harvesting in the harvest stage, the transfer to the packing site, its passage through it and also during the transport that takes it to its final destination. This produces a decline in its quality and, therefore, economic damage. In strawberry studies, 51% of this damage occurs in the harvesting operation, 32% in transport to the destination market and only 17% in packing [3]. Several researchers have proposed that the mechanical damage in the fruit is given by three factors: The impact, the vibration and the pressure by the weight of the fruit [4,5]. In the case of cherries, the processes that cause the loss of quality are three times faster at 20 ${}^{\circ }$C than at 10 ${}^{\circ }$C, and hence the importance of placing them quickly in cooling chambers when the harvest temperature is high. For its part, Sanford et al. [6] states that the damage in cranberries is a product of mechanical damage and storage temperature. However, according to authors in [7], the definition of mechanical damage is not completely clear and the authors give different definitions. However, there is a general coincidence: Mechanical damage is caused by one or more types of load (shock or load pressure) [8,9]. In this way, Timm et al. [10] described two different types of mechanical damage during post-harvest handling: (a) Impacts during the process of harvesting, selecting, handling and transporting the fruit; and (b) compression loads during packing or storage lines.

In the post-harvest stage, from the packing site to the destination markets, solutions for the monitoring of agricultural products mainly aim at measuring the humidity and temperature within the transport vehicles. These studies incorporate global positioning systems (GPSs) to pinpoint the location of the transport vehicle, general packet radio services (GPRSs) for the communication of the transport vehicle with the monitoring station and the use of radio frequency identification (RFID) for the identification of the products [11]. Some of these technological solutions are commercially available, guaranteeing that the fruit will be transported under appropriate conditions and following the schedule committed by the company. Logistics during the harvest stage, however, are much more difficult to monitor, due to the relatively small number of personnel assigned to supervision tasks compared to the large number of workers associated.

Ampatzidis et al. [12,13] address the problem of the integration of technologies in fruit orchards, with the inclusion of radio frequency identification (RFID) technology and bar codes in harvest bins and orchard trees of cherries. With the help of an electronic scale and differential global positioning systems (DGPSs) in the tractors that transport the harvest bins, measurements of the weight associated with the harvested fruit are efficiently acquired. This allows to establish productivity indicators of the orchards and harvest personnel. This research effort recognizes that the weighing process, by means of an electronic scale on the tractor that transports the recollection bins, increases the loading time by almost 33%, and proposes to solve this issue by acquiring these measurements directly at harvesting baskets automatically. However, this strategy does not take into account the fact that losses in the logistics chain, and the management of resources, are also related to the harvest personnel.

Some orchards in Chile use bar code technologies to identify the harvest baskets and the personnel that collect the fruit, by recording the number of baskets used by each collector and the time between each full basket delivery. However, the collected data does not provide information about the damage underwent by the fruit during this process [7]. Moreover, this approach does not consider relevant variables such as temperature and vibrations during harvesting, which can produce mechanical damage to the fruit, accelerating the dehydration process and finally decreasing the shelf life of the product.

Galeas et al. [14] presented a low complexity prototype of a basket with built-in sensors of weight, vibrations and temperature. The main result was the identification of transition stages of the basket based only on the signals acquired. The transition stages were shown to be useful to identify the time that the basket is in fruit picking, in waiting for transportation, in transportation to the packing site and in packing. Some of the sensors used were low-cost and not invasive, such as the IMUs and temperature sensors, because they are based on microelectromechanical (MEM) devices; however, the weight sensor requires supporting the strain gauges with mechanical parts inside the harvest basket, impacting directly on the fabrication costs. This design needs improvement by finding a way of removing the mechanical component without compromising the functionality of the basket. To achieve this development it is required to identify the harvest’s time transitions without using the fruit’s weight. The lengths of the time that elapsed in each one of the harvest phases are useful for avoiding high temperatures during prolonged periods of waiting time.

Time transition identification is a challenging problem; in particular for settling this because, as is shown in Galeas [14], the harvest phases go sequentially, and due to the data provided being gathered from low-cost instrumentation, the problem is well suited for the use of the hidden Markov chains methods and the work of Rabiner [15]; for this particular problem the Viterbi algorithm is one of the best suited.

In this regard, the objective of this article is to propose a novel monitoring system for berry harvesting processes that is solely based on the use of temperature and vibration sensors to perform inferences and estimate the most likely trajectory and switch times between each harvesting process stage. The obtained trajectory estimate will be then used to compute a potential damage indicator for the fruit in terms both of the registered temperature and vibration energy.

## 2. Theoretical Background

### 2.1. Markov Chains

The proposed harvesting stage detection algorithm is built on the assumption that this sequence of stages can be modeled as a hidden Markov model (HMM). Before going into the details that support this assumption, though, it is important to define the concept of a first-order Markov process. A Markov process is a stochastic process that satisfies the Markov property (sometimes characterized as “memorylessness”), that basically states that one can make predictions for the future of the process based solely on its present state, i.e., conditional on the present state of the system, its future and past states are independent [15].

A first-order Markov chain is a particular case of a Markov process [15]. To define it properly, let us consider a system such that its condition at any time instant can be characterized by a finite set of states $S_{1},S_{2},\cdots ,S_{N}$. At any time, this system can change its operational condition in time (i.e., the system makes a transition from one “state” to another), with transition probabilities that are conditional on the current state. We denote the state transition times as t=1,⋯, and the state at any given time *t* as $q_{t}$ [15].

The probabilistic model for system state transitions for the specific case of a discrete first-order Markov chain is completely described by the state transition matrix *A* and the initial state probability distribution Π, where:

(1) $$P\left[q_{t}=S_{j}|q_{t-1}=S_{i}\right]=a_{ij},\;\;1\leq i,j\leq N,$$

and where:

- $a_{ij}\geq 0$
- $\sum_{j=1}^{N}a_{ij}=1$.

This Markov process is denominated “observable” since the system output is a state that can be directly measured. The probability of a given sequence can be computed in this case using the following straightforward procedure:

(2) $$\begin{array}{cc} P(O=\left\{S_{n_{0}},S_{n_{1}},\cdots ,S_{n_{l}}\right\}|Model) & =P\left[S_{n_{0}}\right]\cdot P\left[S_{n_{1}}|S_{n_{0}}\right]\cdots P\left[S_{n_{l}}|S_{n_{l-1}}\right]\\ & =\pi_{0}\left(n_{0}\right)\cdot a_{n_{0}n_{1}}\cdots a_{n_{l-1}n_{l}} \end{array}$$

### 2.2. Hidden Markov Models (HMMs)

In many practical cases, the system state cannot be directly measured and must be estimated. These cases can be well characterized through the concept of hidden Markov models (HMMs). The adjective “hidden” refers to the state sequence through which the model passes, not to the parameters of the model; the model is still referred to as a hidden Markov model even if these parameters are known exactly. Measurements are linked to the system states via a conditional probability density function. As a consequence, the resulting model has two sources of uncertainty that affect the inference problem: (i) Hidden state dynamics and (ii) measurement noise [15].

A discrete HMM is characterized by the following parameters:

- N: Number of states. The set of possible states can be denoted by $S=\{s_{1},\cdots ,S_{N}\}$. The state of the system at time *t* is denoted $q_{t}$.
- M: Number of measurements associated with each state. Each measurement corresponds to a physical outcome from the system that can be acquired using the appropriate sensors.
- The transition probability distribution between system states $A=\{a_{ij}\}$ where:
  (3) $$P\left[q_{t}=S_{j}|q_{t-1}=S_{i}\right]=a_{ij},\;\;1\leq i,j\leq N$$
- The measurement probability distribution conditional of the state *j*, $B=\{b_{j}\left(k\right)\}$:
  (4) $$\begin{array}{cc} b_{j}\left(k\right)=P[O_{k}|q_{t}=S_{j}], & 1\leq j\leq N\\ & 1\leq k\leq M \end{array}$$
- The initial probability distribution of system states π, where:
  (5) $$\pi_{i}=P\left[q_{1}=S_{i}\right],\;1\leq i,j\leq N$$

Considering all of the above, for convenience the following compact notation is typically used to denote the entire set of parameters that characterizes the HMM:

(6) λ=(A,B,π)

**Figure 1:**
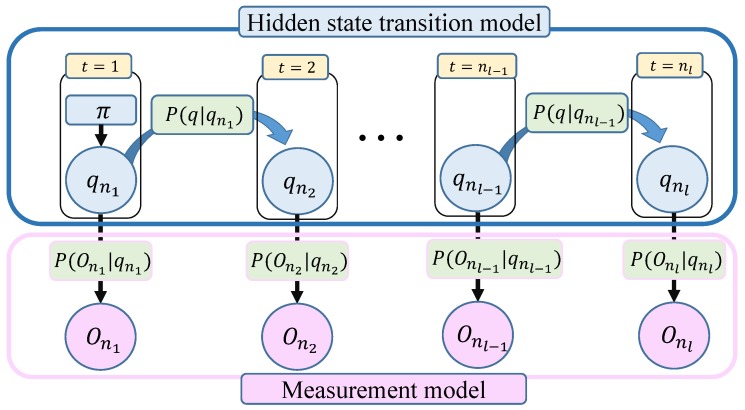
Graphic representation of a discrete hidden Markov model (HMM).

A realization of an HMM is graphically depicted in Figure 1. It is important to note that part of the system dynamics are hidden to the observer (“hidden evolution model”). In addition, in an HMM there is an observational model, which is conditional on the state trajectory. The objective in an inference problem based on HMMs is to estimate the sequence of hidden states $S=\{s_{1},\cdots ,S_{N}\}$ conditional on a set of system measurements $O=\{O_{1},\cdots ,O_{N}\}$ [15].

### 2.3. Viterbi Algorithm

The Viterbi algorithm (VA) [16,17,18] was proposed as a solution to the decoding of convolutional codes by Andrew J. Viterbi in 1967. This algorithm had a great impact in the fields of communications and signal processing, extending its influence to other domains such as the problem of state estimation in stochastic nonlinear systems. The Viterbi algorithm (VA) aims at finding the optimal estimate for a sequence of hidden states (called the Viterbi path) in an HMM, conditional on a set of system measurements. This task is achieved using a dynamic programming formulation, where the inference problem is divided in a series of small stages (indexed by the time associated with each observation). At each stage, the VA finds the optimal value for the state within the sequence, and it continues the analysis to the next stage in an inductive manner. Formally speaking, to find the optimal sequence of hidden states $Q^{*}=\left\{q_{1}^{*}q_{2}^{*}\cdots q_{T}^{*}\right\}$ in a realization of an HMM, conditional on a sequence of system measurements $O=\{O_{1}O_{2}\cdots O_{T}\}$, the following variable is defined: [15,16]:

(7) $$\delta_{t}\left(i\right)=\max_{q_{1},q_{2},\cdots ,q_{t-1}}P\left[q_{1},q_{2},\cdots ,q_{t-1},O_{1}O_{2}\cdots O_{T}|\lambda \right],$$

where $\delta_{t}\left(i\right)$ is the most likely path for the HMM at time *t*, considering the first *t* observations and the state $S_{i}$ as terminal conditions. By induction, it is possible to write:

(8) $$\delta_{t+1}\left(i\right)=\left[\max_{i}\delta_{t}\left(i\right)a_{ij}\right]\cdot B_{j}\left(O_{t+1}\right).$$

As a result, the inference problem is solved by using the pseudo-code shown in Appendix A.

## 3. Materials and Methods

### 3.1. The Blueberry Harvesting Process

**Figure 2:**
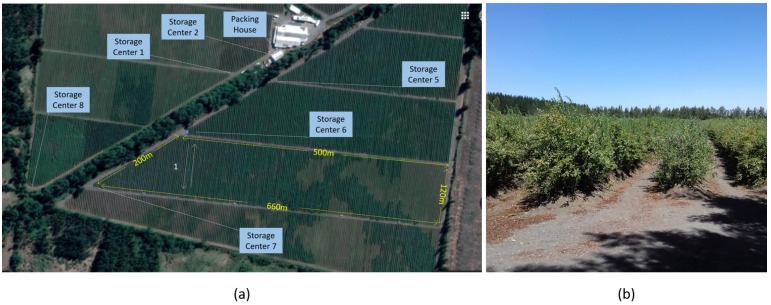
Blueberry orchard in Yungay, Chile. (**a**) Top view showing storage centers and packing house. (**b**) Land view from storage center 6.

The experiment is carried out inside the Boldo S.A. orchard. This orchard has 50 hectares planted with blueberries and is located in Yungay, Chile, in coordinates lat: −37.1149584, long: −72.1973101. As shown in Figure 2, its packing site is located at the center of the garden and there are roads that divide the plantation of blueberries into 3 sectors and each of these sectors is divided into 7 sub-sectors for the irrigation process. Each sector has different varieties, including: Duke, Rabiteye, Brightwell, Tifblue, O’neal and Brigitta.

The process of picking fresh blueberries is done manually and begins by assigning a crew of collectors in each sector of the garden. Figure 2a shows a top view of the garden showing the orchard, the packing house and the storage centers (places provided with shade to temporarily store the boxes previous to delivery at the local packing site), while Figure 2b shows a ground view from the storage center number 6. The collectors walk through the orchard arranged in rows of approximately 120 meters in length, provided with a plastic box of 3.5 L hung from the neck by a harness. Sometimes the collector must walk to the other side of the set of rows (approximately 600 m) to start picking berries. The harvesting process has a duration of 20 to 50 min, depending on the experience of the harvester, the proximity of the rows to be collected, and how much fruit is in the bushes. Once the plastic box is filled, the collector return to the first storage center. In the reception center, another worker increases the count of the number of boxes harvested by the collector and records the time when it was received. Finally, the collector is provided with an empty box to restart the picking process.

The boxes full of fruit are stored in this storage center awaiting a truck with a trailer to take them to the local packing site. After arriving at the packing site the net weight of fruit picked is recorded using an electronic scale. Then, the filled boxes enter into the packing site throughout a freezing tunnel to lower the temperature of the berries. Inside the packing site, the boxes are emptied over a classification table and the boxes are recycled to begin a new harvest cycle.

### 3.2. A Modular Distributed Monitoring System for the Harvesting Process: The “Smartbin”

The proposed system was developed using a harvest basket of 3.5 L, which incorporated two components:

A main device installed on one of its sides, which contains a SODAQ Autonomo device (SODAQ, Hilversum, The Netherlands), a real time clock, temperature sensors and an inertial unit (IMU) to measure the vibrations of the harvest basket and detect the shocks suffered by it.

A false base sustained with a load cell, to measure at all times the weight carried by the basket.

**Figure 3:**
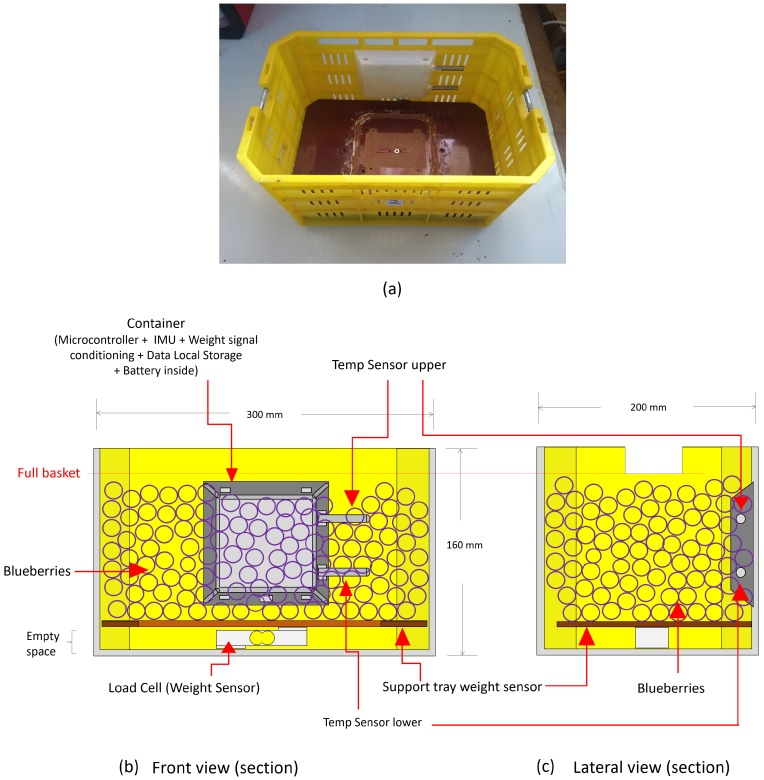
Basket conditioned to on line measure of weight, two temperatures and accelerations; (**a**) basket view, (**b**) schematic front view and (**c**) schematic lateral view.

The SODAQ Autonomo uses an Atmel SAMD21J18 processor, with 256 kb of Flash memory, 32 kb of SRAM memory and a 32-bit processor running at 48 MHz. In addition, it has a socket for the use of a micro SD card, which allows internal storage of the data. A real time clock (DS1307) with the time and date was added to this device, information that is attached to all captured data. The IMU used is based on the MPU-9250 chip with an accelerometer, a gyroscope and a 3-axis magnetometer. The unit also has two temperature sensors based on the digital device DS18B20 (Dallas Semiconductor, Dallas, TX, USA), the accuracy of which is 0.5 ${}^{\circ }$C. These temperature sensors protrude like two tubes of the main device, glued to one of the internal walls of the harvest box to measure the temperature of the berries at two heights, 6 cm and 10 cm from the base of the box (see Figure 3). The load cell located in the false base of the box is connected to an analog/digital converter HX711 which in turn is connected to the SODAQ Autonomo device. The system was provided with a Li-ion battery of 2300 mAh/3.7 V for its energy autonomy, which was estimated at 30 h of continuous operation.

Figure 3a presents a photograph of the final prototype, while Figure 3b,c shows the front section and the lateral section of the prototype. The false base, or support tray for the weight sensor, left a useless space at the bottom of the basket because the tray must relay over the load cell to carry on the weighting procedure. The two temperature probes give information about the fruit’s temperature inside the basket and close to the surface only when the IoT basket is full. The difference between the temperature measures can be used to detect when the fruit level has reached the two positions where the temperature probes are installed.

This main device works as a remote collection unit and as a data logger, transmitting wireless and storing all the data collected in an microSD card installed in the “SODAQ Autonomo" device, with two types of records, one that is written every 100 ms with the measurements of the IMU, date and time, and another that is written every 15 s with measurements of temperature, weight, voltage of the battery, date and time. These time measurements are taken to identify faults in the system, and correlate the tests with the events that occurred during the day.

### 3.3. Data Acquisition Campaign

Data from the experimental campaign was acquired using 5 “smartbins” in an experimental set up carried out during one day in the middle of the harvest season of blueberries in the “El Boldo” orchard (see Figure 2). Each one of the 5 “smartbins” was used in two consecutive harvest cycles during the day of the experiment. As a result, it was possible to record 10 complete harvesting cycles (each cycle finishes with the bin returned to the hands of the picker after being emptied). The structure of the acquired dataset can be summarized as follows:

- Temp_1: Temperature measurement acquired every 15 s using a sensor that is located near the bottom of the bin.
- Temp_2: Temperature measurement acquired every 15 s using a sensor that is located near one of the four the external edges of the bin.
- $Acc_{x}$: Acceleration measurement in *x*-axis acquired ten times per second with an IMU located inside the bin.
- $Acc_{y}$: Acceleration measurement in *y*-axis acquired ten times per second with an IMU located inside the bin.
- $Acc_{z}$: Acceleration measurement in *z*-axis acquired ten times per second with an IMU located inside the bin.
- Weight: Net weight of the “smartbin” acquired every 15 s with a sensor that is located at the bottom of the bin.

In terms of nomenclature, and for all practical purposes, each harvesting cycle was labelled using the following format: $N_{i}c_{j}$, where i=1,2,3,4,5 refers to the ith bin and j=1,2 indicates the number of the recorded cycle for that specific bin.

In eight of these cycles $N_{i}c_{j}$, where i=2,3,4,5, j=1,2 were used as training data, while two cycles were used for validation purposes ($N_{1}c_{1}$ and $N_{1}c_{2}$, both corresponding to the 1st bin).

To avoid loss of information, the signals were processed using raw values.

### 3.4. Proposed Methodology for Online Harvesting Stage Detection

The proposed methodology uses the Viterbi algorithm to perform inferences of datasets and estimate the most likely state trajectory in the harvesting process. Indeed, this case study allows to define a finite number of possible “states” (each one associated with one stage of the harvesting procedure), making it a perfect candidate for the implementation of inference schemes based on the assumption of HMMs. The set of observations *O* incorporate data from IMUs and temperature sensors. While the entire process has six “states” that can be identified (picking, waiting, transport (full bin), cooling, emptying and transport (empty bin)), only four of them are hereby considered. The latter since only the first four states are critical in terms of quantifying the potential damage to the fruit during the harvesting procedure (the “emptying” state is fully automated, and afterwards the bin is empty). These states are:

(1) “Picking”(S1): The pickers, provided with a 3.5 L plastic box hung around the neck by a harness, cover the orchard prepared in rows approximately 100 m long. Picking lasts 20 to 40 min per box, depending on the picker’s experience and the volume of fruit on the shrubs. During this stage it is possible to measure high energy vibration signals and high temperatures.
(2) “Wait” (S2): When the box is full, the picker goes to the storage center (shaded area), where he/she delivers the box for counting. The full boxes remain at the warehouse waiting for the tractor–trailer to take them to the local packing area.
(3) “Transport” (full bin) (S3): The tractor–trailer transports full boxes from the warehouse to the local packing area.
(4) “Cooling” (freezer tunnel) (S4): The fruit is admitted to packing via a conveyor table, where a cooling system lowers its temperature using a freezing tunnel.

Considering all of the above, an HMM is trained for this case study using eight harvesting cycles $N_{i}c_{j}$, where i=2,3,4,5, j=1,2. Ground truth for the transition times between states in training (and also validation) data was defined by incorporating information acquired from the weight sensor that is located at the bottom of the “smartbin”. Weight sensor measurements allow to simplify the detection of state transitions because they help to determine the moment when the “picking” stage is over (bin weight measurements stabilize at a constant value, a condition that can be tested by a basic hypothesis testing procedure), as well as the exact moment when the bin is emptied. Conditional to the latter transition times, it is possible to discriminate the “cooling” stage just by detecting sudden drops in temperature measurements, while “wait” and “transport” stages can be identified since they differ significantly in terms of the associated energy in the IMU signal.

The challenge behind the proposed method for state transition detection is to avoid the usage of weight measurements altogether (except, as in this case study, for purposes of determining ground truth transition times in training data). The latter since it would be preferable and significantly cheaper to eliminate this weight sensor from the original design of the “smartbin”. For this purpose, an HMM is conceived to describe the transition between the stages of the harvesting process, where the observation space is solely determined by the following sensor information:

(1) Inertial measurement unit (IMU): Data acquired by the IMU. A simple pre-processing algorithm is implemented to complement this information with an average of the total energy in the vibration signal every 15 s over the time window containing the last 15 s of measurements.
  (9) $$IMU\_Energy_{t}=\sum_{j=t-14}^{t}acc_{x}{\left(j\right)}^{2}+acc_{y}{\left(j\right)}^{2}+acc_{z}{\left(j\right)}^{2}$$
(2) Temperature measurements: Besides the information provided by sensors Temp_1 and Temp_2, a simple pre-processing algorithm is implemented to measure the difference in readings between both temperature sensors.
  (10) Delta_T(t)=Temp_2(t)−Temp_1(t)

Considering all of the above, and following the maximum likelihood estimation procedure explained in [15] to determine the coefficients of state transition matrices in an HMM, it is possible to state that the harvesting process can be characterized by the following matrices:

(11) $$\begin{array}{c} A=\left[\begin{array}{cccc} 0.9153 & 0.0847 & 0.0000 & 0.0000\\ 0.0000 & 0.8169 & 0.1831 & 0.0000\\ 0.0000 & 0.0000 & 0.6966 & 0.3034\\ 0.0000 & 0.0000 & 0.0000 & 1.0000 \end{array}\right] \end{array}$$

(12) $$\begin{array}{c} \pi =\left[\begin{array}{cccc} 1 & 0 & 0 & 0 \end{array}\right] \end{array}$$

where *A* is obtained by computing the expected residence time on each state in the training dataset [15]. In this case, π is known since the HMM is always initialized in state S1 (“picking”). The characterization of the entire process using an HMM allows to use the Viterbi algorithm for state transition time detection purposes.

### 3.5. Proposed Methodology for Fruit Damage Indicator

A natural byproduct associated with the implementation of the Viterbi algorithm for estimation of the most likely state path is that it is also possible to detect start and end times for each of the different stages of the berry harvesting process. These start and end times become critical information to characterize the potential damage accumulated during “picking”, “waiting” and ‘transport” stages since during that lapse the fruit in the bin is exposed to a higher level of vibrations and elevated temperatures. As established by [3,6,19,20,21], long exposures to high temperatures and high quantities of dissipated energy contribute to early damage to the fruit. Inspired by this fact, this research effort has proposed the following damage indicator to assess the potential damage incurred by the fruit during the harvesting process.

(13) $$\begin{array}{c} DamageIndicator=\frac{1}{10^{5}}\cdot \left(\sum_{i=0}^{T_{S4}}Temp\_2_{i}+\sum_{i=0}^{T_{S4}}IMU\_Energy_{i}\right) \end{array}$$

where IMU_Energy is a variable that indicates the energy associated with the vibration signal recorded by sensors in the bin during a 15 s sliding window. $T_{S4}$ corresponds to the moment in which the Viterbi algorithm detects a transition from states S3 to S4, measured in seconds. The temporal reference t=0 is established to be synchronized with the start of the “picking” stage.

The proposed indicator for potential fruit damage offers robustness against disturbances in estimates of transition times, since it solely depends on $T_{S4}$ for all practical purposes. Indeed, $T_{S4}$ determines the start of the “cooling” stage and thus it is expected to observe at that time simultaneous (and sudden) drops in readings of sensors Temp_1 and Temp_2, while the energy in the vibration signal should be small compared to the “picking” and “transport” stages. This evidence anticipates that errors in the estimate of $T_{S4}$ should be negligible in comparison to the total time allotted for the harvesting cycle, and therefore the value of the proposed damage indicator, which depends on the overall accumulation of stress on the fruit, should not exhibit significant changes to its value.

## 4. Obtained Results in Experimental Campaign

**Figure 4:**
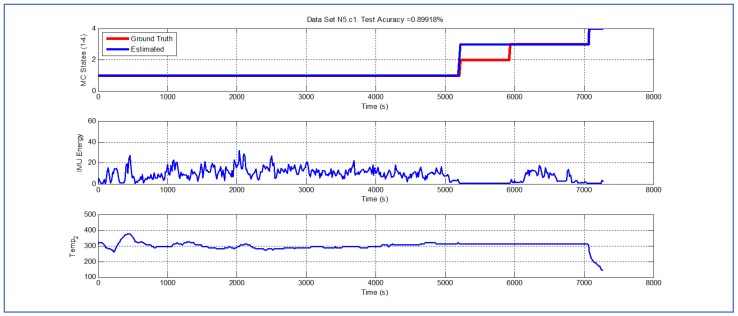
Detection of berry harvesting stages. dataset $N_{5}c_{1}$.

**Figure 5:**
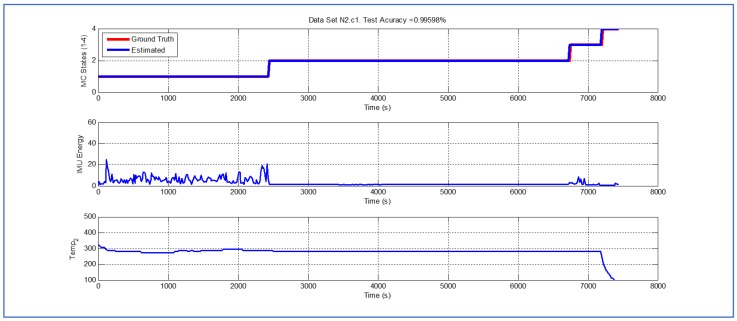
Detection of berry harvesting stages. Dataset $N_{2}c_{1}$.

**Figure 6:**
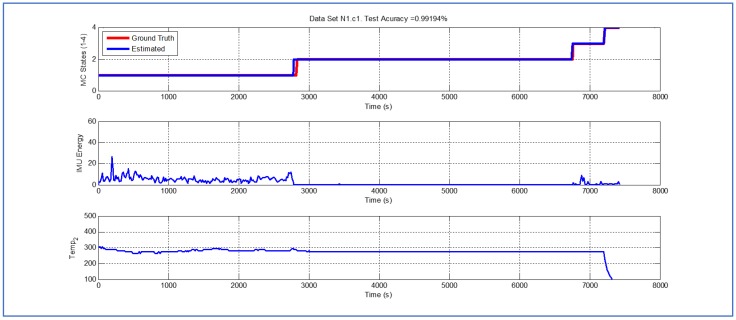
Detection of berry harvesting stages. Dataset $N_{1}c_{1}$.

**Figure 7:**
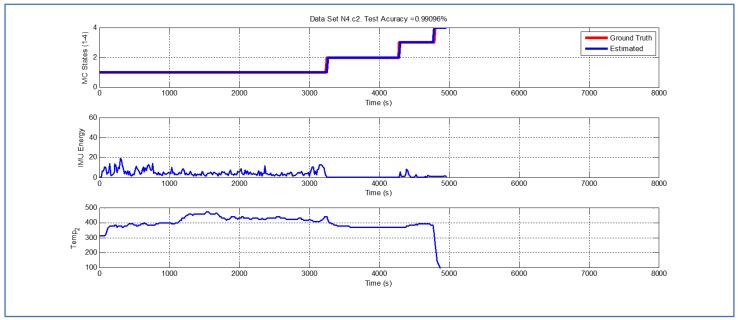
Detection of berry harvesting stages. Dataset $N_{4}c_{2}$.

**Figure 8:**
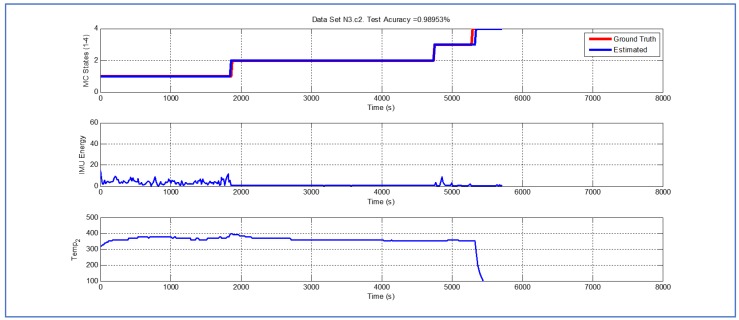
Detection of berry harvesting stages. Dataset $N_{3}c_{2}$.

**Figure 9:**
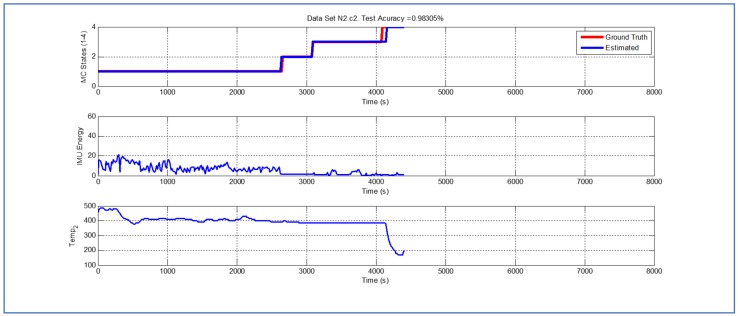
Detection of berry harvesting stages. Dataset $N_{2}c_{2}$.

**Figure 10:**
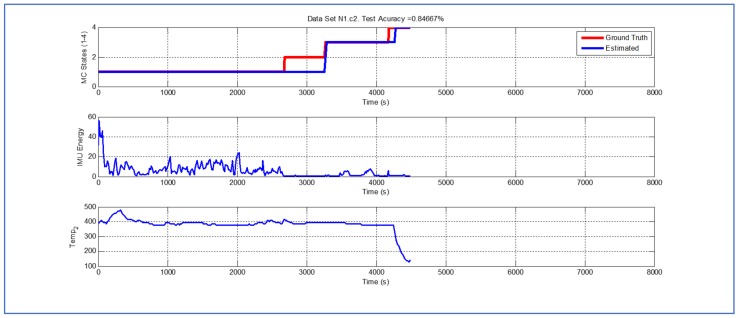
Detection of berry harvesting stages. Dataset $N_{1}c_{2}$.

**Figure 11:**
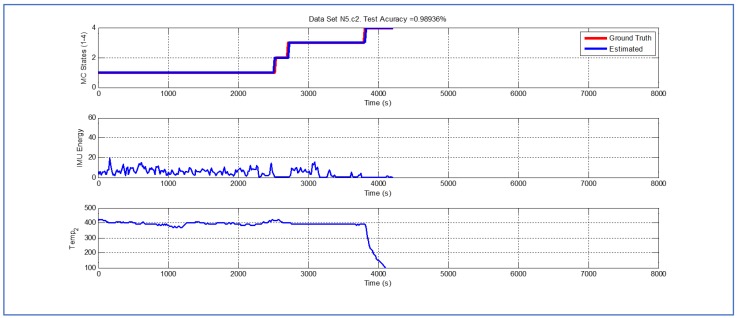
Detection of berry harvesting stages. Dataset $N_{5}c_{2}$.

**Figure 12:**
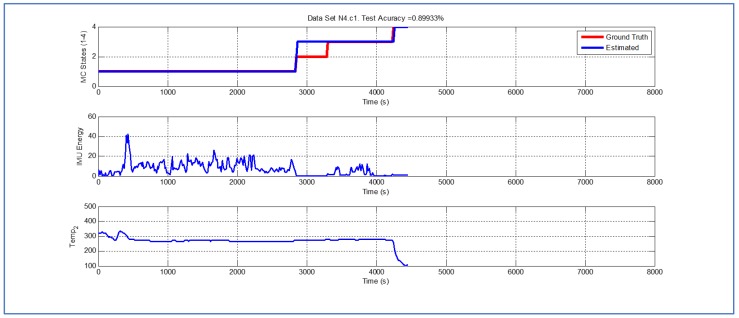
Detection of berry harvesting stages. Dataset $N_{4}c_{1}$.

**Figure 13:**
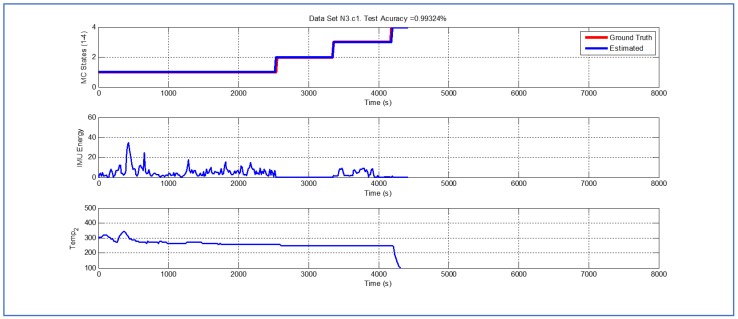
Detection of berry harvesting stages. Dataset $N_{3}c_{1}$.

Table 1 and Figure 4, Figure 5, Figure 6, Figure 7, Figure 8, Figure 9, Figure 10, Figure 11, Figure 12 and Figure 13 show the results obtained when applying the proposed scheme for the harvest stage recognition and potential fruit damage assessment on actual field data from an experimental campaign. Each figure consists of three graphs that help to understand the manner in which the proposed algorithm interprets the acquired data. The first graph shows the performance exhibited by the Viterbi algorithm in the detection of transitions between each one of the first four stages of the harvesting process: ”Picking”, ”wait”, ”transport” and ”cooling”. The second graph shows the energy of the IMU signal (averaged over a 15 s sliding window), and finally the third graph on each figure shows the temperature registered on the second temperature sensor inside the bin. Figure 4, Figure 5, Figure 6, Figure 7, Figure 8, Figure 9, Figure 10, Figure 11, Figure 12 and Figure 13 are sorted in terms of the one that represents the most potential fruit damage to the one that is more innocuous. Given the structure of the proposed damage indicator, both the time of exposure of the fruit at ambient temperature (principally at states S1–S3) and cumulative energy of vibration signals (principally at state S1) have critical influence on the assessment of potential damage.

Figure 4 illustrates a case where the potential fruit damage is the greatest. One of the reasons that explain this statement is the fact that in this cycle the fruit was exposed to relatively high ambient temperature for a lengthy lapse of time. Moreover, both during the “picking” and “transport” stages, the energy of the IMU accelerometer signal is significant, indicating that the fruit in the bin could have been shaken excessively. It is important to note that the Viterbi algorithm in this case fails to detect the transition between states S1 and S2 (overall efficacy in detection in this dataset is 89.918%). While this issue affects the tractability of the bin in the system, it does not have an impact on the assessment of potential fruit damage since the transition to S4 (“cooling stage”) is perfectly detected. Figure 5 and Figure 6 illustrate a case where potential fruit damage is significantly high. While the same concepts explained in the previous case also apply here, it is important to note that the energy associated with the vibration signal is lower than in the case of Figure 4. Moreover, please note that the performance of the Viterbi algorithm is high (overall efficacy in detection in these datasets is 99.396%), exhibiting a negligible delay in the detection of the transition between S1 and S2 in dataset $N_{1}c_{1}$, the latter being used for purposes of validating the proposed approach.

While temperature associated with the data shown in Figure 7, Figure 8 and Figure 9 is higher than their predecessors, the lapse of time where the fruit was exposed to ambient temperature is considerably smaller. In both cases, there is a small delay in the estimate of parameter $T_{S4}$, but the performance of the Viterbi algorithm is still beyond 98.95%.

Validation dataset $N_{1}c_{2}$ (Figure 10) is the one where the Viterbi algorithm exhibits the lowest performance (overall efficacy in detection in these datasets is 84.667%). Nevertheless, even in this case, the error associated with the estimate of parameter $T_{S4}$ is 90 s, which represents 2% in a dataset that records 4485 s of operation.

Last but not least, Figure 11, Figure 12 and Figure 13 exhibit analogous performances in terms of the accuracy of the Viterbi algorithm. Interestingly, in terms of potential fruit damage, the most innocuous dataset corresponds to one where the ambient temperature was low, and where the harvesting cycle lasted less than 4425 s.

## 5. Conclusions

This article proposes a monitoring system for berry harvesting solely based on the use of temperature and vibration sensors. The monitoring system assumes a characterization of the process in terms of a hidden Markov model and uses the Viterbi algorithm to perform inferences and estimate the most likely state trajectory.

The obtained state trajectory estimate is then used to compute a potential damage indicator for the fruit in terms both of the registered temperature and vibration energy, with an overall average efficacy in detection for validation datasets of 91.937%, while errors in the estimates of the moment at which the bin reaches the cooling stage were not larger than 2%, a fact that validates the proposed damage indicator as a robust feature for characterization of the potential degradation in the quality of the fruit when used in conjunction with the Viterbi algorithm for purposes of estimating the value of $T_{S4}$.

More importantly, the proposed procedure proves to be equivalent in terms of the effectiveness in the characterization of the stages of the harvesting process to other alternatives found in the literature, but significantly more efficient since it does not require information about the weight of the bin in which the fruit is collected to identify the different stages of the harvesting process and determine indicators that could help to assess if this harvesting process is being performed normally. It seems that the Viterbi algorithm is a complex solution for this problem but it is inexpensive to include those procedures in the software running on the microprocessor of the “smartbins”, avoiding the need to measure weight and consequently disregarding the strain gauges and the mechanical parts needed to support them. The fact that it is possible to dispense with the utilization of weight sensors in the design of “smartbins”, replacing them with more advanced signal processing tools, has a significant economic impact in terms of the penetration of these monitoring devices in the agricultural market as a right solution for some of the problems that the industry has faced over these years. The information provided by these “smartbins” is helpful to support decisions with economic significance for the producers, such as infrastructure investment, locations of the storage centers, schedules for transportation between the storage centers and the packing house and quantity and training of the personnel working as fruit pickers.

## Figures and Tables

**Table 1:** Experimental campaign: Harvesting cycles ordered in terms of potential fruit damage.

Harvesting Cycle ($N_{i}c_{j}$: ${\mathrm{bin}}_{i}$, ${\mathrm{cycle}}_{j}$)	Damage Index
$N_{5}c_{1}$	1.4762
$N_{2}c_{1}$	1.3710
$N_{1}c_{1}$	1.3386
$N_{4}c_{2}$	1.3017
$N_{3}c_{2}$	1.2905
$N_{2}c_{2}$	1.1239
$N_{1}c_{2}$	1.1119
$N_{5}c_{2}$	1.0218
$N_{4}c_{1}$	0.8002
$N_{3}c_{1}$	0.7484

## Appendix A

The Pseudo-code for the implementation of the Viterbi algorithm is:

**Algorithm 1** Viterbi Algorithm (λ=(A,B,π),O)	
**Inputs:** λ=(A,B,π),O	
**Output:** $Q^{*}=\left\{q_{1}^{*},q_{2}^{*},\cdots ,q_{T}^{*}\right\}$	
1: **for** i=1,⋯,N**do**	▹ Initialization
2: $\delta_{1}\left(i\right)=\pi_{i}b_{i}\left(O_{1}\right)$	
3: $\psi_{1}\left(i\right)=0$	
4: **for** j=1,⋯,N, t=2,⋯,T **do**	▹ Recursion
5: $\delta_{t}\left(j\right)=\underset{1\leq i\leq N}{max}\left[\delta_{t-1}\left(i\right)a_{ij}\right]\cdot B_{j}\left(O_{t}\right)$	
6: $\psi_{t}\left(i\right)=\underset{1\leq i\leq N}{argmax}\left[\delta_{t-1}\left(i\right)a_{ij}\right]$	
7: $P^{*}=\underset{1\leq i\leq N}{max}\left[\delta_{T}\left(i\right)\right]$	
8: $q_{T}^{*}=\underset{1\leq i\leq N}{argmax}\left[\delta_{T}\left(i\right)\right]$	
9: **for** t=T−1,T−2,⋯,1 **do**	▹ Reconstruction of state sequence
10: $q_{t}^{*}=\psi_{t+1}\left(q_{t+1}^{*}\right)$	
11: **return** $Q^{*}$	
